# An image-based metal artifact reduction technique utilizing forward projection in computed tomography

**DOI:** 10.1007/s12194-024-00790-1

**Published:** 2024-03-28

**Authors:** Katsuhiro Ichikawa, Hiroki Kawashima, Tadanori Takata

**Affiliations:** 1 Faculty of Health Sciences, Institute of Medical, Pharmaceutical and Health Sciences, Kanazawa University, 5-11-80 Kodatsuno, Kanazawa, 920-0942 Japan; 2 Department of Diagnostic Radiology, Kanazawa University Hospital, 13-1 Takara-machi, Kanazawa, 920-8641 Japan

**Keywords:** Computed tomography, Metal artifact, Forward projection, Projection data, Parallel computation

## Abstract

The projection data generated via the forward projection of a computed tomography (CT) image (FP-data) have useful potentials in cases where only image data are available. However, there is a question of whether the FP-data generated from an image severely corrupted by metal artifacts can be used for the metal artifact reduction (MAR). The aim of this study was to investigate the feasibility of a MAR technique using FP-data by comparing its performance with that of a conventional robust MAR using projection data normalization (NMARconv). The NMAR_conv_ was modified to make use of FP-data (FPNMAR). A graphics processing unit was used to reduce the time required to generate FP-data and subsequent processes. The performances of FPNMAR and NMAR_conv_ were quantitatively compared using a normalized artifact index (AI_n_) for two cases each of hip prosthesis and dental fillings. Several clinical CT images with metal artifacts were processed by FPNMAR. The AI_n_ values of FPNMAR and NMAR_conv_ were not significantly different from each other, showing almost the same performance between these two techniques. For all the clinical cases tested, FPNMAR significantly reduced the metal artifacts; thereby, the images of the soft tissues and bones obscured by the artifacts were notably recovered. The computation time per image was ~ 56 ms. FPNMAR, which can be applied to CT images without accessing the projection data, exhibited almost the same performance as that of NMAR_conv_, while consuming significantly shorter processing time. This capability testifies the potential of FPNMAR for wider use in clinical settings.

## Introduction

The computer tomography (CT) image is reconstructed from the projection data acquired from an X-ray scanner. Prior to reconstruction, the projection data are processed in accordance with the purpose, e.g., to remedy image quality insufficiencies such as an inaccuracy of CT number due to beam hardening or to compensate for increased noise due to a low radiation dose or metal artifacts. The projection data can also be obtained through numerical forward projection (FP) applied to the CT image data. For example, iterative reconstruction (IR) techniques, which have recently been used to reduce radiation dose in CT acquisitions [1, 2], employ FP to compare the projection data with its original.

The projection data generated by FP (hereafter called “FP-data”) have useful potentials for improving CT image quality when only image data are available, because it contains X-ray attenuation information similar to that in the original projection data. However, this potential has not been exploited widely so far. For example, to our best knowledge, only one paper reports its use in reducing the streak artifacts caused by low photon counts [3]. This limited use seems to be attributable to the large amount of computing time, and therefore cost, required for FP and the subsequent filter back projection (FBP) to reconstruct a CT image. In recent decades, however, the performance of graphics processing units (GPUs) capable of highly parallel computation has increased remarkably, while their prices have dropped dramatically. As a result, the time and cost of computation required for the above processing have come down to a level within the reach of most medical institutions, making approaches based on FP-data much more realistic and feasible.

Another possible use of FP is the reduction of metal artifact (MAR). In MAR, image-based approaches have not been sufficiently investigated to date, whereas those based on original projection data have been well investigated [4–6]. In fact, several MAR applications have already been implemented in clinical CT systems [7]. The convolutional neural networks (CNNs) have been utilized in some studies; however, investigations were mainly performed using phantom simulations [8, 9] and deformations and/or disappearing of anatomical structures newly caused by the CNN process were observed in the resultant images [10], which suggested further investigations using the CNN for clinical images. Since the metal artifacts frequently cause severe image alternations such as streaks, distortions and banding and their effects vary depending on the conditions of the metal such as density, location, size, and the number of pieces. Thus, MAR based on training the CNN is still presenting a considerable challenge [10]. Given this difficulty, MAR using FP-data is considered a useful option that does not require image collection for CNN training. However, there is a question of whether the FP-data generated from an image severely corrupted by metal artifacts can be used in place of the original projection data, which has not been investigated to date.

The purpose of this study was, therefore, to investigate whether the FP-data can be used for MAR by comparing its performance with that of a conventional MAR technique using projection data normalization, which are thus far known to be robust.

## Materials and methods

### MAR procedure

**Fig. 1:**
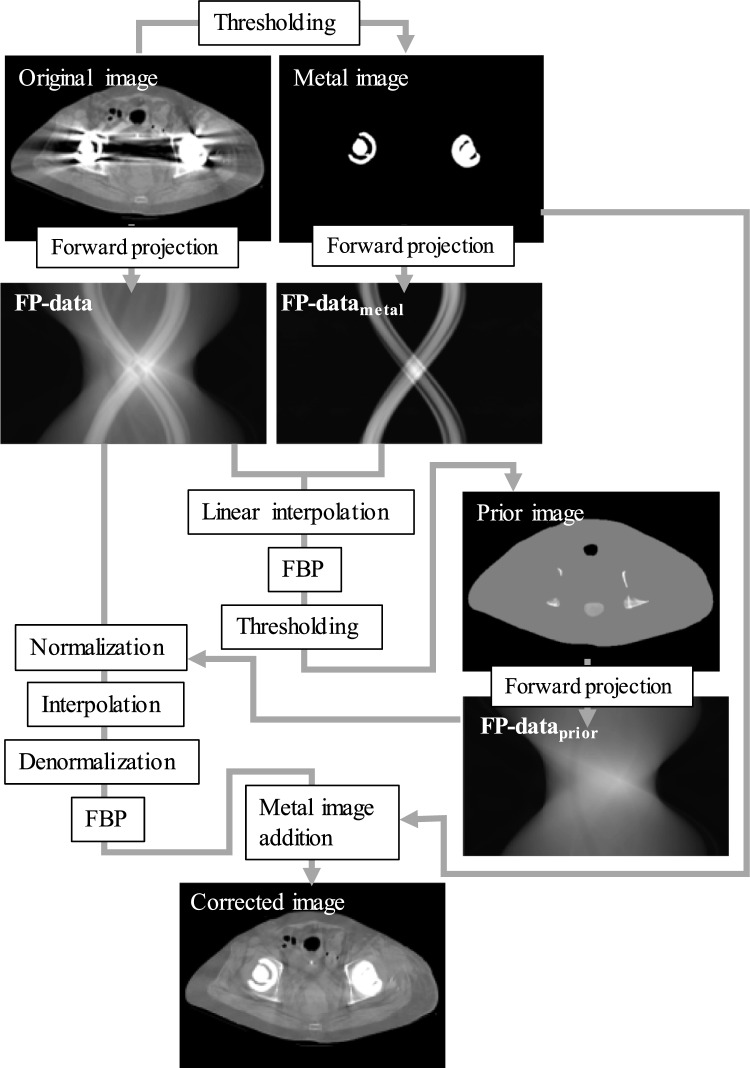
Outline of the metal artifact reduction (MAR) technique using projection data generated via a forward projection (FP-data). A conventional MAR technique based on projection data normalization (NMAR_conv_) was modified to this version that utilizes FP-data (FPNMAR)

A conventional MAR technique based on projection data normalization (NMAR_conv_) [4] was modified to handle FP-data (FPNMAR). Figure 1 shows an outline of the FPNMAR technique. First, the original CT image with metal artifacts was forward-projected (FP-data). Second, a metal-only image was extracted from the original CT image using simple thresholding; then the image was again forward-projected to produce a projection of the metal trace (FP-data_metal_). The metal trace was used to identify the regions for inpainting. Third, simple linear interpolation was used to obtain the first MAR image, from which a prior image that had plain and smooth texture was made using thresholding: the air regions were set at − 1000 Hounsfield unit (HU) and the soft tissue parts at 0 HU, while the bone pixels kept their values. The prior image was forward-projected to produce FP-data_prior_; then, the FP-data were divided (normalized) by the FP-data_prior_ and, after linear interpolation, denormalized. Finally, an MAR image was reconstructed from the denormalized projection, followed by an addition of the metal-only image. NMAR_conv_ was performed using the original projection data instead of the FP-data and the fan-beam geometry of the CT scanner we used in this study for the forward and back projections.

### Forward projection

**Fig. 2:**
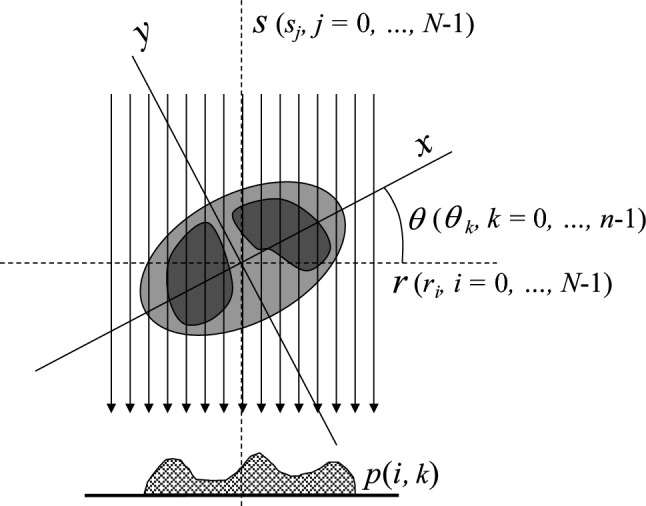
Coordinates in numerical parallel forward projection to generate the FP-data. For each discrete angle *θ*_*k*_, along the ray projection at a discrete position *r*_*i*_, sampled data at *s*_*j*_ were accumulated to *p*(*i*, *k*). For the sampling, bicubic interpolation was used

The processing technique used in forward projection was the same as that used in reducing streak artifacts by utilizing FP-data as reported by our laboratory team [3]. The report demonstrated that the CT images reconstructed from filtered FP-data had almost the same spatial resolution as that of the original images and had precisely the same CT numbers. Figure 2 shows the relationship between the coordinates in the FP and those in the rotated CT image data. We employed parallel-beam projection instead of fan-beam projection, which is commonly used in clinical CT systems, because the theory of CT reconstruction assures that fan-beam projection data can be converted to parallel-beam data by a known conversion algorithm [11]. As shown in this figure, we employed a rotation-based method in which a projection data array remains fixed and the image is rotated through interpolation [12]. At each ray position of *r*_*i*_ (*i* = 0, …, *N − *1), data sampling at *s*_*j*_ (*j* = 0, …, *N − *1) was repeated and accumulated to *p*(*i*, *k*) at each projection angle *θ*_*k*_ (*k* = 0, …, *n − *1) as follows:

1 $$p(i,k) = \sum\limits_{j = 0}^{N - 1} {q(i,j)}$$

2 $$x(i,j) = r_{i} \cos (\theta_{k} ) - s_{j} \sin (\theta_{k} )$$

3 $$y(i,j) = r_{i} \sin (\theta_{k} ) + s_{j} \cos (\theta_{k} ),$$

where *x* and *y* are the *x*- and *y*-sampling positions in the original CT image data *c*(*l*, *m*) (*l* = 0, …, 511, *m* = 0, …, 511), respectively, and *q*(*i*, *j*) is the data sampled from *c*. Bicubic interpolation was used for the sampling with an *α* parameter of − 0.5 because this method can produce a smaller spatial resolution loss compared with the bilinear technique [13]. The sampling interval was set at half the pixel pitch of the CT image; thus, 1024 points were required (*N* = 1024) for each of the *r*_*i*_ and *s*_*j*_ positions. A total of 800 projections (*n* = 800) were made at intervals of 180°/*n* within 0° ≤ *θ* < 180°. This unit of calculation was performed in parallel for *i* = 0, …, 1023, and *k* = 0, …, 799 using a GPU capable of highly parallel computing (Geforce RTX4070Ti, Micro-Star Int'l, Taiwan) in combination with a 64-bit central processing unit (CPU) (Core i7-12700, Intel Corporation, California, USA). In this way, the total calculation time was notably reduced. The FP-data in a 1024 × 800 matrix were thus obtained; then, the matrix size was increased to 2048 × 800 by padding zeros on both its left side and right side to enable the filtering process in FBP.

### FBP

Filtering in the FBP was performed using a two-dimensional fast Fourier transform (2D-FFT) function that is incorporated in the development environment for the GPU (Compute Unified Device Architecture: CUDA, Nvidia). This function is optimized for the architecture of the GPU and can perform a 2D-FFT for a 2048 × 800 matrix in less than 1 ms. After the filtering, a back projection process was performed as follows. For each pixel location of *x*′(*l*, *m*) and *y*′(*l*, *m*) in an image to be reconstructed, the *r* position *r*_*l*, *m*_ in the filtered projection data at *θ*_*k*_ can be calculated as follows:

4 $$r_{l,m} = x^{\prime}(l,m)\cos (\theta_{k} ) + y^{\prime}(l,m)\sin (\theta_{k} ).$$

The sampling data *τ* (*l*, *m*, *k*) at *r* in the projection data at *θ*_*k*_ were obtained through one-dimensional cubic interpolation with an *α* parameter of − 0.5; then the sampled data were integrated (back-projected) to the pixel *c*′ (*l*, *m*) as follows:

5 $$c^{\prime}(l,m) = \sum\limits_{k = 0}^{n - 1} {\tau (l,m,k)}.$$

These calculations were parallelized for *l* = 0, …, 511, and *m* = 0, …, 511 using the GPU.

### Capriciousness in FPNMAR

The display field of view (DFOV) varies depending on the diagnostic purposes. As a result, there is some capriciousness with FP-data, which affects its performance in MAR. Since metal artifacts frequently spread to areas outside the object, CT number alterations due to spread artifacts are generally ignored when the DFOV includes only the object’s contour. Moreover, when the DFOV is set in a size smaller than the contour, the CT number alterations are further ignored. Naturally, metal artifacts caused by metals outside the DFOV cannot be compensated for; thus, such artifacts are not covered by FPNMAR. While being aware of these problems, we performed the FPNMAR and evaluated its MAR performance.

### Clinical cases

Images of hip prostheses, dental fillings, and coils in brain aneurism coiling in clinical cases scanned with a 64-slice CT scanner (Somatom Definition Edge, Siemens Healthineer, Germany) were processed by the FPNMAR. Two clinical cases each of hip prosthesis and dental filling were selected for a comparison between FPNMAR and NMAR_conv_. These cases are the same ones used in a previous study of our laboratory team [14] that purposed improvement of image texture in the MAR image generated using original projection data. To quantitatively compare the artifact strengths of the two NMAR methods, we measured the normalized artifact index AI_n_, which is defined as follows:

7 $$ \ {{\text{AI}}}_{{\text{n}}}=\frac{\sqrt{{{{\text{SD}}}_{{\text{A}}}}^{2}-{{{\text{SD}}}_{{\text{B}}}}^{2}}}{{{\text{SD}}}_{{\text{B}}}},$$

where SD_A_ denotes the standard deviation (SD) measured of the CT image with metal artifacts; SD_B_ is the SD measured of the CT image with no metal artifacts [15]. The normalization using SD_B_ was adopted because the spatial resolution of the image reconstructed based on the FP-data is slightly different from that of the image reconstructed from the original projection data. The region of interest (ROI) with 20 × 20 pixels for SD_A_ was placed at a central position between both hip joints in the bladder for the hip prosthesis case and the lingua for the dental filling cases. The ROI for SD_B_ was placed in the soft tissue with no visible structures in muscles around the ilium or the oropharynx. Thus, for each case, a single SD_B_ was obtained by averaging the SD values of the ten selected images and was used as the common value for SD_B_ for AI_n_ calculations of the ten consecutive images with metal artifacts. The AI_n_ values were compared between FPNMAR and NMAR_conv_, by means of the Wilcoxon signed-rank test with *p* < 0.05 taken as statistically significant.

This retrospective use of the image and projection data was approved by our institutional review board, and since we performed only additional image reconstruction, the requirement for written informed consent was waived for all the patients.

### Computation time

For FP-data generation, FP-data filtering, back projection, and the total processing in FPNMAR, the computation time was measured for ten CT images selected from those used in this study. For comparison between with and without the GPU, we rewrote the FPNMAR program using normal sequential codes and then measured computation time. The filtering of FP-data was performed using a 2D-FFT function provided in Intel Math Kernel Library 2019. The built-in timer in the Microsoft visual C++ 2013 was used for the measurement.

## Results

### Comparison of FPNMAR and NMAR_conv_

**Fig. 3:**
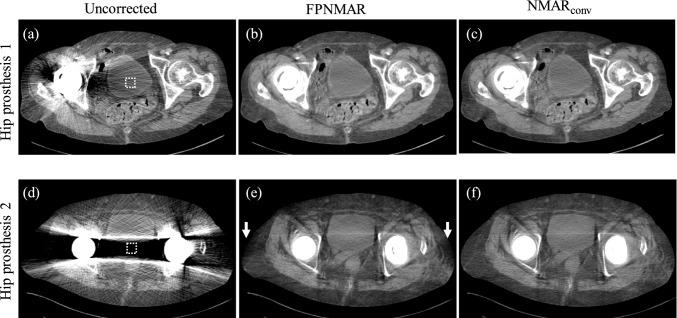
Uncorrected, FPNMAR-processed, and NMAR_conv_-processed images for two cases of hip prosthesis. Arrows indicate portions with a CT number decrease. The window’s width and level were set at 500 HU and 40 HU. The position of region of interest (ROI) for measuring the normalized artifact index (AI_n_) is presented with the dashed box in each uncorrected image

**Fig. 4:**
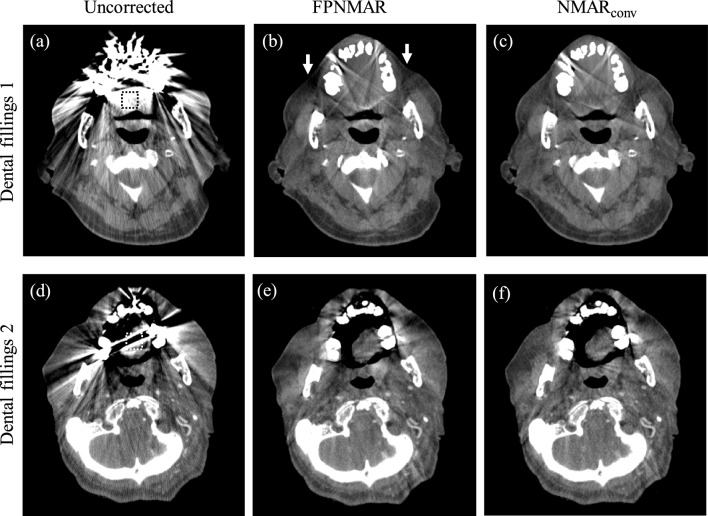
Uncorrected, FPNMAR-processed, and NMAR_conv_-processed images for two cases of dental fillings. Arrows indicate portions with a CT number decrease. The window’s width and level were set at 500 HU and 40 HU. The position of ROI for measuring the AI_n_ is presented with the dashed box in each uncorrected image

Figures 3 and 4 show the uncorrected, FPNMAR-processed, and NMAR_conv_-processed images of hip prostheses and dental fillings, respectively. Table 1 presents the corresponding results of AI_n_. For all the cases of the hip prostheses and dental fillings, FPNMAR reduced the metal artifacts almost as effectively as NMAR_conv_ (there were no significant differences in AI_n_ value between the two). Incidentally, some decreases in CT number were observed with FPNMAR images. For hip prostheses, they occurred on both peripheral sides of the body as shown in Fig. 3e (pointed at by the arrows); for dental fillings, they were observed on the bilateral cheeks as shown in Fig. 4b (pointed at by the arrows).

**Table 1:** Measured normalized artifact indices (AI_n_) for uncorrected, FPNMAR-processed, and NMAR_conv_-processed images and *p* values for comparison between FPNMAR and NMAR_conv_

	Hip prosthesis		Dental fillings	
	Case 1	Case 2	Case 1	Case 2
Uncorrected	2.71 ± 0.35	9.15 ± 1.55	32.12 ± 4.28	43.85 ± 5.25
FPNMAR-processed	0.51 ± 0.06	0.56 ± 0.10	2.52 ± 0.51	2.12 ± 0.45
NMAR-processed	0.46 ± 0.07	0.52 ± 0.07	2.32 ± 0.36	1.92 ± 0.54
*p* value (FPNMAR vs NMAR)	0.325	0.458	0.528	0.274

### FPNMAR clinical images

**Fig. 5:**
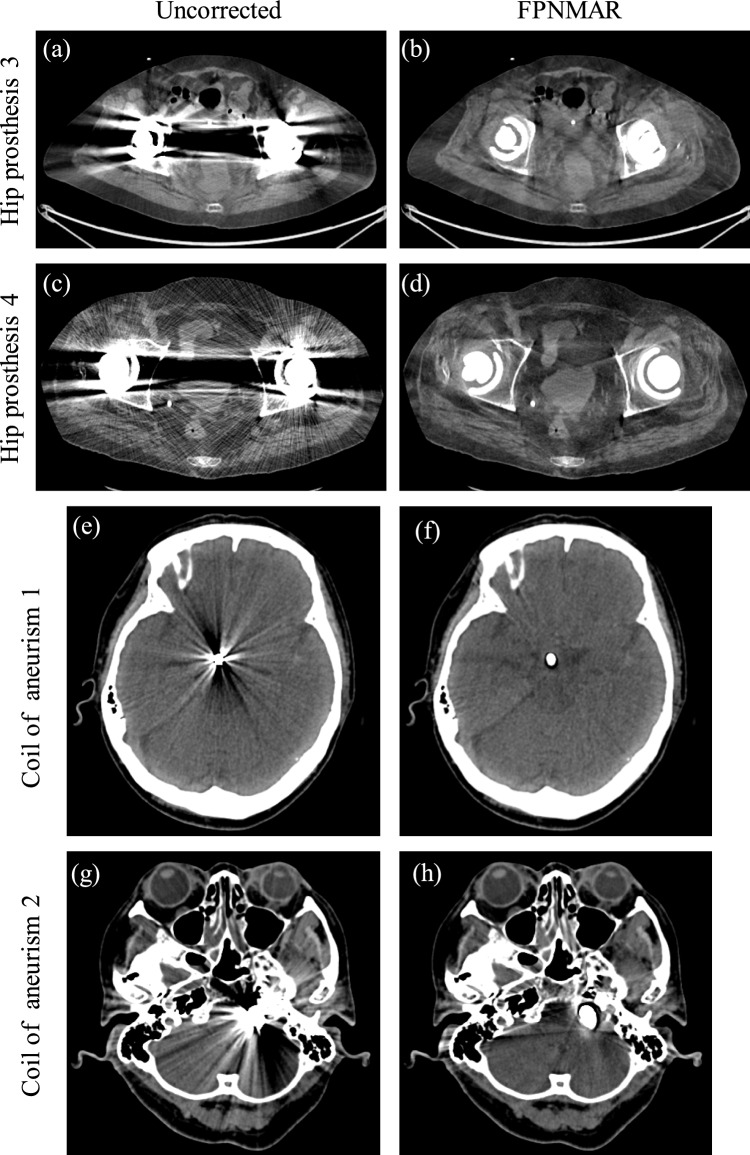
Uncorrected and FPNMAR-processed images of the other two cases of hip prosthesis and two cases with coils for aneurism. The window’s width and level were set at 500 HU and 40 HU; 250 HU and 40 HU, for hip prosthesis and aneurism coiling cases, respectively

**Fig. 6:**
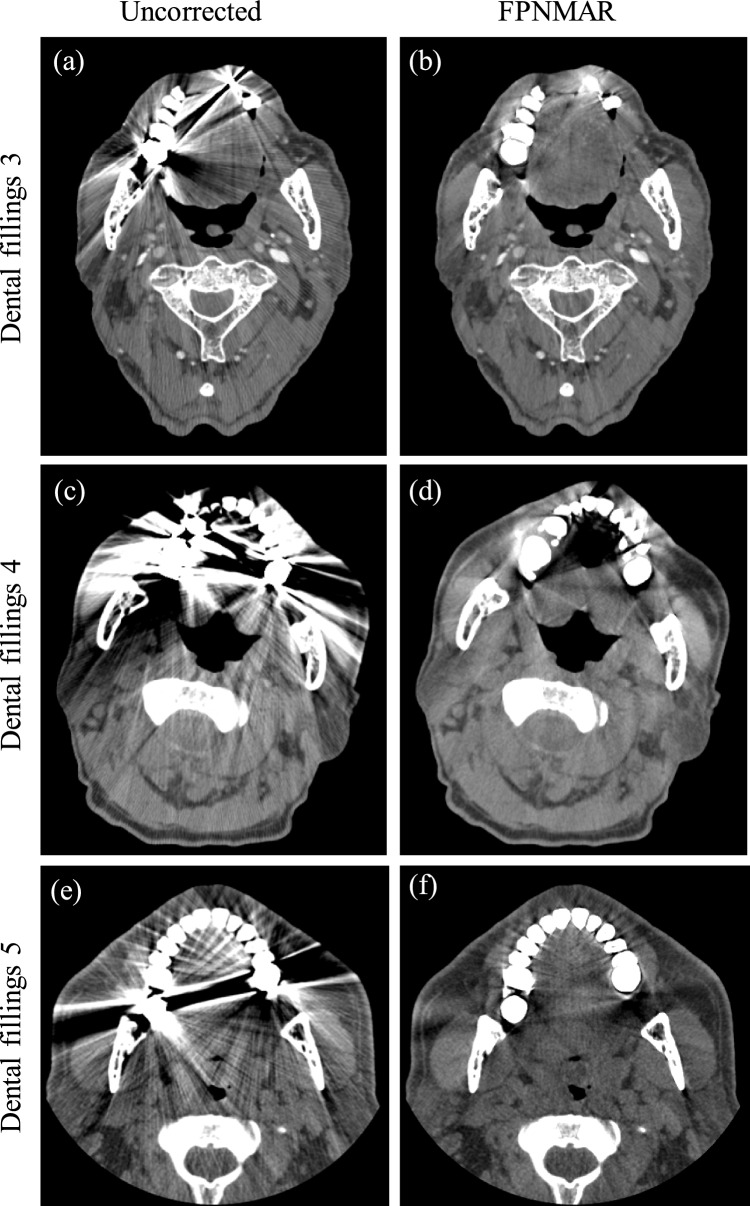
Uncorrected and FPNMAR-processed images of the other three cases of dental fillings. The window’s width and level were set at 500 HU and 40 HU

Figure 5 shows uncorrected and FPNMAR-processed images of clinical cases of hip prosthesis and coils for aneurism. Figure 6 shows uncorrected and FPNMAR-processed images of clinical cases of dental fillings. Severe artifacts were mostly eliminated and soft tissues and bones obscured by the artifacts became visible for all the cases. The CT number decreases were also caused in one of cases of the hip prosthesis (Fig. 5d). One case did not include the entire head contour (Fig. 6e and f); however, the metal artifacts were markedly reduced.

### Computation time

Table 2 shows the measured computation time for FP-data generation, FP-data filtering, back projection, and the total with and without the GPU. Within each item of processing, the computation time did not fluctuate noticeably. With the GPU, we consider the total processing time of about 56 ms sufficiently short. In contrast, we consider the total processing time of about 66 s for the FPNMAR program without the GPU practically almost unacceptable in most clinical settings.

**Table 2:** Averaged computation time of FPNMAR with and without the GPU: FP-data generation, FP-data filtering, back projection, and the total

	FP-data (ms)	Filtering (ms)	Back projection (ms)	Total (ms)
GPU	15.6 ± 0.1	0.4 ± 0.0	1.9 ± 0.0	55.8 ± 0.9
w/o GPU	21,521.8 ± 80.5	35.9 ± 3.8	917.3 ± 10.8	66,553.7 ± 90.5

The entire processing consisted of three runs of FP-data generation, two runs of FP-data filtering, two runs of back projection, and other processes related to the inpainting of the FP-data

## Discussion

We have investigated an MAR technique based on FP-data (FPNMAR), with which artifacts were reduced almost as effectively as with a conventional MAR technique that uses the original projection data (the NMAR_conv_). This observation was endorsed by AI_n_ being almost equal between the FPNMAR and NMAR_conv_ for two clinical cases each of hip prosthesis and dental fillings. For all the clinical cases, FPNMAR noticeably reduced metal artifacts and, as a result, the soft tissues and bones obscured by the artifacts became visible. These results demonstrate that MAR can be achieved with clinical CT images without accessing the original projection data. The parallel computation we applied was notably effective because the calculation time per image was merely about 56 ms, making FPNMAR not only less stressful but also more feasible for clinical use.

In theory, FP-data can be equivalent to the original projection data for CT images adequately reconstructed without conspicuous artifacts, thanks to the complementary relationship between radon transform and inverse radon transform. However, for CT images with severe CT number alterations such as metal artifacts, this equivalency has not been demonstrated to date in studies using actual CT images. The present study demonstrates that FPNMAR can reduce metal artifacts as effectively as NMAR_conv_, thereby pointing at significant potential for wider use of FP-data in clinical settings.

There was some capriciousness in the FP-data of the image portions, which manifested itself as a decrease in CT number and some minor differences in image between the FPNMAR and NMAR_conv_. In most other respects, however, the overall performance of FPNMAR was favorable: with severe CT number alterations of the metal artifacts effectively eliminated, plausible CT number distributions were restored. Furthermore, it is worth noting that a sufficient reduction in artifacts was achieved on CT images reconstructed with a small DFOV not covering the entire contour of the object (Fig. 6e and f). With this small DFOV, although the generated FP-data were remarkably different from the original projection data, it seems to still retain some of the data representing many parts of the artifacts, albeit incompletely. Whereas only one case is presented in this report, we did observe similar effects in other cases with small DFOVs. This fact seems to reflect the complex, or even self-redundant nature of MAR and to reveal an interesting property of FP-data.

In typical clinical settings, MAR techniques cannot always produce correct images in that artifacts are not completely eliminated and the original CT number distributions are not correctly recovered. This is because projection data corrupted by the X-ray opacity of a metal object is replaced by the data estimated by means of various ‘approximation’ techniques. Thus, it is generally recommended that both the uncorrected and MAR images be used in image interpretation [7]. The MAR images processed by FPNMAR in the present study showed some unfavorable CT number changes. This drawback, however, should be considered acceptable, taking into account that MAR images are by nature not always accurate. FPNMAR, therefore, can be considered, along with other MAR techniques, as one of the alternative approaches with high potential to efficiently reducing artifacts.

As mention earlier, MAR techniques based on training the CNN is still presenting a considerable challenge. In contrast, the proposed FPNMAR is efficient in that it does not require the laborious CNN training. Moreover, FPNMAR could potentially be used to generate images for the CNN training.

This study has some limitations. First, only one CT system was used for evaluating the performance of FPNMAR. The strengths and appearances of metal artifacts depend on various factors such as the X-ray quality (e.g., effective energy) and the reconstruction method; thus, the efficacy of FPNMAR should be evaluated using various CT images produced by different systems. Second, we did not try to apply other MAR techniques such as one based on beam hardening correction (BHC), which had been proposed as another way to use the original projection data [6]. Taking into account the close affinity of FP-data with the original projection data of CT images having metal artifacts, as demonstrated in this study, MAR techniques based on BHC may be worth further investigation. Third, no observation study involving radiologists was performed. The influence of FPNMAR on image interpretation should be evaluated.

## Conclusion

The proposed FPNMAR technique applied to CT images without accessing the corresponding projection data reduced metal artifacts as effectively as MAR_conv_, which is based only on the original projection data. For all the clinical cases we examined, this technique not only reduced the metal artifacts but also helped recover the soft tissue and bone images that had been obscured by the artifacts. As an additional benefit, it significantly reduced the processing time down to approximately 56 ms, thanks to the GPU capable of highly parallel computation, revealing its potential for wider use in clinical settings.

## Data Availability

The data from this study are available from the corresponding author upon reasonable request.
